# Expecting the best or fearing the worst: Discrepancies between self‐rated health and frailty in an ageing Irish population

**DOI:** 10.1111/bjhp.12585

**Published:** 2022-02-11

**Authors:** Bill Calvey, Joanna McHugh Power, Rebecca Maguire

**Affiliations:** ^1^ Hamilton Institute Maynooth University Co. Kildare Ireland; ^2^ Department of Psychology Maynooth University Co. Kildare Ireland

**Keywords:** frailty index, health congruence, health discrepancy, older adults, self‐rated health

## Abstract

**Objectives:**

Ageing populations have the propensity to rate their health status more inaccurately than their younger counterparts. As a result, we (1) devised a metric which categorized older adults into groups based on the discrepancy between their self‐rated health (SRH) and Frailty Index (FI) scores, and (2) investigated which factors predict group membership.

**Design:**

A cross‐sectional design was employed using data from The Irish Longitudinal Study of Ageing (TILDA).

**Methods:**

A health asymmetry metric was derived: this categorized 6907 participants (aged 50+ years) into three groups: ‘health pessimistic’ where participants underestimated their healthiness, ‘health realistic’ where participants accurately assessed their health, and ‘health optimistic’ where participants overestimated their healthiness. A multinomial logistic regression modelled the ability of a set of sociodemographic, psychosocial, and health behaviour variables in predicting membership of these categories.

**Results:**

A significant proportion of the study population were categorized as ‘health realistic’ (~69%). The prevalence rates of health optimistic individuals increased in older age groups, and conversely, health pessimistic rates decreased in older age groups. Most notably, psychosocial factors significantly predicted being health pessimistic: such as anxiety (OR = 1.03), loneliness (OR = 1.04), and decreased social connectedness (OR = 0.87). However, less clear sociodemographic, psychosocial, and health behaviour associations were found for being health optimistic.

**Conclusion:**

Health asymmetry is a useful method of identifying at‐risk individuals for inaccurate SRH. The ability of this metric to predict clinical mental health outcomes should be investigated.

**Statement of contribution**

***What is already known on this subject?***

- Self‐rated health (SRH) is a reasonably reliable proxy for objective health status.
- However, the reliability of SRH comes into question in ageing populations, where trends become less defined.
- Considerable individual variability exists with SRH estimations: some individuals overestimate their healthiness whilst others underestimate their healthiness.

***What does this study add?***

- A novel metric is presented for the identification of inaccurate health status perceptions: categorizing ageing individuals into ‘health pessimistic’, ‘health realistic’, and ‘health optimistic’ categories, by contrasting their SRH and frailty scores.
- Sociodemographic, health behaviours, and psychosocial factors are useful in predicting health pessimistic individuals but are less useful in predicting health optimistic individuals.
- Health asymmetry is a useful way to identify at‐risk individuals for inaccurate self‐rated health estimations and its ability to predict clinical health outcomes should be investigated further.

## Background

Self‐rated health (SRH) is a multi‐dimensional and complex health construct, which relates to the subjective perception of an individual’s own health status. This can simply be measured by asking an individual how healthy they think they are, with five options, generally ranging from ‘poor’ to ‘excellent’. Interestingly, SRH can reveal a considerable amount of information about an individual’s disease, functional status (Meng, Xie, & Zhang, 2014), and mental health (French, Sargent‐Cox, & Luszcz, 2012). SRH is therefore considered a reliable proxy for objective health status and has been shown to independently predict mortality (Bath, 2003; Falk et al., 2017; Idler, 2003). As a result, SRH is now considered an interdisciplinary measure ubiquitously deployed in many fields of research, including psychology, epidemiology, and economics. Despite its usefulness however, the subjective health measure is undoubtedly complex and still poorly understood – even after extensive research. Most notably, considerable individual variability exists in the estimation of SRH (Sokol, Ennett, Gottfredson, & Halpern, 2017), which leads to some difficulties using SRH as a health measure.

Gaps in understanding of SRH are not due to a lack of empirical evidence, but rather vagueness around what SRH actually measures (Jylhä, 2009). This includes inconsistent operational definitions among other issues. For example, there has been contestation over whether SRH solely measures physical health or a more global perspective on an individual’s health. Krause and Jay (1994) note that, in response to an SRH item, some participants base their rating on specific health problems they suffer from, whilst others think more generally in terms of their health behaviours and functionality. This highlights the importance for consistency and accuracy in phrasing and response options when collecting SRH ratings (Cullati et al., 2020). The WHO (1996) recommended that SRH responses included five worded responses, preferably ranging from ‘very good’ to ‘very bad’. Few changes have been recommended since, though the phrasing of SRH has still varied internationally. For example, responses in some European studies have ranged from ‘very good’ to ‘bad’, with other variations of this too (Jylhä, Guralnik, Ferrucci, Jokela, & Heikkinen, 1998; Robine & Jagger, 2003). Another explanation for the lack of understanding surrounding SRH is reported by Layes, Asada, and Kephart (2012): they purport that a latent process exists with SRH. They argue that cognitive or emotional reporting tendencies such as optimism and pessimism could trigger measurement error and bias in the accuracy of SRH, particularly in those aged 80+. Consequently, such biases along with other measurement intricacies and individual differences may result in unexplained discrepancies between an individual’s SRH and objective health status.

Comparing the accuracy of an individual’s SRH to their objective health status is not a straightforward process. Given the multi‐dimensional nature of health, a gold‐standard objective health scale is unrealistic. Previous studies have operationalized objective health as the amount of disease diagnoses a patient has received, combined with measures of functional capacity, such as Instrumental Activities of Daily Living (IADLs) and Basic Activities of Daily Living (Araújo, Teixeira, Ribeiro, & Paúl, 2018). An issue with such measures, however, is that health is more complex than just disease and functional status. Other aspects of health, such as cognitive and mental health, may be ignored. Similarly, much research in economics has merely focused on singular measures of objective health such as hypertension (Johnston, Propper, & Shields, 2009; Suziedelyte & Johar, 2013), rather than an index or more extensive measures of objective health.

### The frailty index as a potential measure of objective health in ageing populations

One potential measure that may be used to estimate objective health status is the Frailty Index (FI), which is commonly deployed in ageing populations (Mitnitski, Mogilner, & Rockwood, 2001). Frailty has been defined as the susceptibility to decreased reserve, decreased response to stressors, and reduced functionality (O’Halloran, Finucane, Savva, Robertson, & Kenny, 2014), and has also been recognized as a pertinent and clinically relevant geriatric condition (van Kan, Rolland, Morley, & Vellas, 2008). The FI measures global health‐related structures and covers a range of measures of health, such as cognitive, functional, physical health, and illness diagnoses (Searle, Mitnitski, Gahbauer, Gill, & Rockwood, 2008). For example, the FI can include health deficits such as whether an individual has had joint replacement surgery, or whether they experience chronic pain. Given its global health‐related structure, the FI has the potential to be interpreted as a health measure in older patients (Rockwood et al., 2014). The application of FI as a potential health indicator is also suitable in the context of an older population (Wuorela et al., 2020), as it forms a holistic indication of a participant’s frailty and relative functionality, physical, and cognitive health status.

### Predicting inaccurate estimations of self‐rated health

Nielsen (2016) argues that, when rating their own health status, individuals not only assess their current health but also anticipate severe health outcomes which may occur in the future. This may lead to unfounded fears and anxieties around health in some, who may be categorized as ‘health pessimistic’. Essentially, such individuals may be considered to be fearing the worst for their health. Conversely, it is also possible that a cohort of individuals may have an overly hopeful view and are expecting the best in terms of their health, being ‘health optimistic’. While considerable research has been conducted on predictors of SRH generally in terms of sociodemographic, psychosocial, and health behaviour variables (Chow et al., 2018; Svedberg, Bardage, Sandin, & Pedersen, 2006; Vingilis et al., 2002), little is known about the factors that may predict SRH inaccuracies – this will be addressed in this study.

Age is the main risk factor for decline of SRH. A steady decline of SRH is noticed from mid‐to‐late life, whilst being an independent predictor of mortality (Idler & Benyamini, 1997). However, some counter‐intuitive trends in SRH have been noted where older individuals may underestimate their health decline (Henchoz, Cavalli, & Girardin, 2008). This suggests that health discrepancies between SRH and objective health may be more prevalent in older age, and a greater understanding of the predictors of these is merited. Other sociodemographic factors such as marital status are predictive of lower SRH, perhaps due to married individuals sometimes being less isolated and socially restricted (Meadows & Arber, 2015). Aspects of work life are often included in models of SRH decline. Verity et al. (2018) found that employees who work more hours per week are more likely to be categorized as the ‘worried well’ – where individuals possess health concerns about illnesses that are typically absent. Other sociodemographic factors – such as having a close family member who suffers from an illness – are generally known to increase health anxiety levels, leading to an increase of the utilization of health care services (Bilani et al., 2019). Ultimately, this may have implications for health discrepancies.

Additionally, there are psychosocial factors which can be linked to SRH. Most notably, an anxiety diagnosis, in particular health anxiety, is strongly associated with low levels of SRH (Lodin et al., 2019). It might therefore be expected that higher anxiety levels would be predictive of more inaccurate health ratings. Other psychosocial factors, including loneliness and social connectedness, may similarly be indicative of inaccurate SRH. For example, in their longitudinal investigation into the implications of loneliness on SRH, Nummela, Seppänen, and Uutela (2011) found that no or low experience of loneliness was highly predictive of good SRH. Lower levels of loneliness yielded slower SRH decline, which indicates that loneliness is associated with the decline of SRH. Within the SRH literature, what constitutes as good/bad SRH or high/low SRH tends to fluctuate from study to study. However, for consistency, we consider ‘poor’ or ‘fair’ SRH responses to be bad or lower levels of SRH, and we consider ‘good’, ‘very good’, or ‘excellent’ SRH responses as being generally good or higher levels of SRH.

Finally, a collection of health behaviours are repeatedly associated with SRH, particularly in individuals who suffer from chronic pain (Reyes‐Gibby, Aday, & Cleeland, 2002) and diseases (Yang et al., 2021). Persistent smoking is associated with extremely low SRH levels (Wang, Ho, Lo, Lai, & Lam, 2012). Interestingly, while alcohol consumption has been associated with suboptimal SRH levels, a linear relationship is rarely obtained between the two. Instead, often a ‘J‐shaped’ relationship is noted with suboptimal SRH being more frequent in non‐drinkers and binge drinkers, than in moderate drinkers (Grønbæk et al., 1999; Theobald, Johansson, & Engfeldt, 2003; Van Dijk, Toet, & Verdurmen, 2004). A potential explanation for this is that the benefits of moderate drinking may be artificially increased by confounding variables such as education, socioeconomic and marital status, social network and psychological health (Emberson & Bennett, 2006; Fillmore et al., 1998). Additionally, the SRH literature in relation to alcohol consumption remains contradictory in nature, as more recent research has refuted an association between consumption and low SRH (Frisher et al., 2015). Regardless, the daily actions which people consciously undertake have the propensity to be indicative of how accurately they self‐rate their own health.

### Developing a health asymmetry metric

This study aims to create a new metric for the identification of inaccurate health ratings in ageing populations, based on discrepancies between SRH and FI scores. Using data from the Irish Longitudinal Study of Ageing (TILDA), we create a categorical health asymmetry variable, with the following health categories: health pessimistic, where SRH levels are considerably lower than FI scores, health realistic, where individuals accurately assess their own health status and health optimistic, where an individual’s SRH score is considerably higher than their FI score. Additionally, given the lack of multivariate models which have accounted for such discrepancies between health measures, a secondary aim of this study is to conduct a multinomial logistic regression to assess whether a collection of sociodemographic, psychosocial, and health behaviour variables can predict group membership within this health asymmetry categorization.

## Method

### Participants and design

This study used a nationally representative and longitudinal dataset called The Irish Longitudinal Study of Ageing (TILDA), which collates social, economic, and health data from older adults, resident in the Republic of Ireland. This cross‐sectional study draws on the data from Wave 1 (collected between 2009 and 2010), of which 6907 independently living and ageing adults were included in analyses. All participants provided informed consent prior to their participation in TILDA – the project was ethically approved and assessed by the ethics board (Kenny et al., 2010).

### Measures

#### Self‐rated health

SRH was measured on a 5‐point scale, with the responses: ‘excellent’, ‘very good’, ‘good’, ‘fair’, and ‘poor’. Participants were asked how they would rate their health generally, in terms of one of the above responses.

#### Frailty index

A number of health measures collected within the TILDA study were used to compile a unique FI, following guidelines from Searle et al. (2008), including aspects of functional health, physical health, cognitive health, and disease prevalence. Specifically, the FI is computed through the combination of health deficits across these domains (see Table 1). The items in the index remain unweighted, as long as they cover each fundamental aspect of health and frailty, assuming that the frailty scores increase over time. Inclusion of a health deficit is warranted if the health deficit becomes more prevalent with age and does not saturate too early (e.g., reduced eyesight). Each health deficit is computed into a binary variable: in this study, they were labelled as either 1 (deficit not observed yet) or 2 (deficit observed). For example, the measure of chronic pain within this study was dichotomized into whether the participant experienced chronic pain (=2) or not (=1). Continuous variables were converted in a similar manner, whilst being informed by the relevant literature. For example, results from the mini mental state examination were computed as follows: a score of less than 10 indicates severe dementia (=2), a score between 10 and 17 indicates moderate dementia (=1.75), a score between 18 and 20 indicates a diagnosis of mild dementia (=1.5), a score between 21 and 23 reveals a diagnosis of mild cognitive impairment (=1.25), and a score of +24 implies no cognitive impairment (=1), as indicated by Cullen et al. (2005). Overall, each binary deficit is computed together to reveal a whole Frailty Index score; a higher score implies a more frail individual.

**Table 1:** The 26 health deficits computed into the Frailty Index (FI): including functional outcomes of health, disease prevalence, cognitive, and physical health measures

Functional HEALTH	Physical health	Cognitive health	Disease prevalence
IADLs (11 items)	Pain	MMSE	Cancer
Joint replacements	Polypharmacy	Memory Complaints	Hypertension
Healthcare utilization	Supplement Intake		Heart Attack
	Illness‐related weight loss		CHF Diabetes
			Arthritis
			Chronic lung disease

#### Creation of a health asymmetry variable

In order to establish the discrepancy between an individual’s SRH and FI score, a health asymmetry metric was created – a categorial variable with three categories. This was derived in a similar manner to the derivation of ‘social asymmetry’ metrics (McHugh, Kenny, Lawlor, Steptoe, & Kee, 2017; Power, Sjöberg, Kee, Kenny, & Lawlor, 2019) and to similar metrics used to describe premorbid and cognitive functioning (Benke, 2011; Bondi et al., 2008). Firstly, since SRH ratings and FI scores were not measured on similar scales, both were standardized. SRH ratings were then subtracted from FI scores, giving rise to a discrepancy score for each participant. The standard deviation of this discrepancy score was used to determine the cut‐off points for this new categorical variable. Individuals whose discrepancy scores were 1 standard deviation below the mean were categorised as health pessimistic, since their SRH score was considerably lower than their FI score. Participants with a discrepancy score of within 1 standard deviation of the mean were categorized as health realistic, as their SRH and FI scores were relatively consistent with each other. Finally, individuals with a discrepancy score that was 1 standard deviation above the mean were considered health optimistic, as their SRH ratings were higher than their FI score. As is noted in previous asymmetry metrics, the derivation of a new categorical variable from continuous variables can reduce statistical power; however, it is a beneficial way to categorize at‐risk individuals (McHugh et al., 2017).

#### Multivariate model predictors

Other measures from the TILDA dataset were included as predictor variables in a multivariate model to predict membership in the health asymmetry categorizations. These variables were categorized into three main types: sociodemographic, psychosocial, and health behaviour variables (see Table 2). Gender, marital status, educational attainment, work status, relative with a cancer diagnosis or other serious illness, smoking status, vigorous exercise level, sleep quality, and cancer screening participation were all measured as categorical variables, measured as either binary or with multiple responses. The remaining variables were continuous: anxiety was measured using the Hospital Anxiety and Depression scale (HADs) (Zigmond & Snaith, 1983); loneliness was measured using the UCLA loneliness scale (Russell, 1996); social connectedness was measured using a derived variable which accounted for whether an individual was a member of church, was married or living with a partner, was a member of a non‐religious organization, and had at least one close relative. Alcohol consumption was measured based on how many days a week an individual would consume alcohol.

**Table 2:** The sociodemographic, psychosocial, and health behaviour variables to be analysed in the multivariate model

Sociodemographic	Psychosocial	Health behaviours
Sex	Intimate Relationship	Smoking Status
Marital status	Loneliness	Alcohol Consumption
Education level	Social Connectedness	Vigorous Exercise
Hours of work per week	Anxiety	Sleep Quality
Work status		Cancer Screening Attendance
Relative w/ cancer diagnosis		
Relative w/ other serious illness		

### Data analysis

All data analyses were completed in R Studio. There were few missing datapoints across the entire dataset (2.34%), though a significant amount of this missingness was contained within four of the variables to be entered into the multivariate model (loneliness, alcohol consumption, intimate relationship, and social connectedness). Multiple imputation was conducted to fill in these missing values. The R package ‘Multiple Imputation by Chained Equations’ (MICE) was utilized to conduct the imputation (Van Buuren & Groothuis‐Oudshoorn, 2011). Continuous data were imputed using predictive mean matching, whilst categorical data were imputed using polytomous regression.

Prior to modelling, preliminary analyses were run to ensure that there was no violation of model assumptions, particularly multicollinearity. A multinomial logistic regression was conducted to predict group membership of the categorical criterion health asymmetry variable, based on a set of sociodemographic, psychosocial, and health behaviour variables. The multinomial logistic regression model can be generalized as follows:

$$\log\left[\frac{p(X)}{1‐p(X)}\right]\;=\;\alpha_{0}+\beta_{1}x_{1\;}+\;\beta_{2}x_{2\;}+\cdots +\;\beta_{k}x_{k}$$

where *X* = ($X_{1\;}\cdots \;X_{k}$) are k predictors. Maximum likelihood estimation was utilized in the model to estimate the coefficients $\beta_{1}\cdots \;\beta_{k}$ for k predictors. The quantity *p*(*X*)/1 – *p*(*X*), which is exponentiated, is the log odds: the probability of a respondent being categorized in a health category, in relation to the reference category. The log odds or ‘logit’ were interpreted to assess how the sociodemographic, psychosocial, and health behaviour variables predict categorization within the health asymmetry groups: health pessimistic, health realistic, and health optimistic. Probabilities of group membership were calculated in relation to a reference category, which was set as health realistic in this model. The logit model was conducted using the ‘nnet’ package in R (Venables & Ripley, 2002).

## Results

### Health asymmetry categorizations

**Figure 1:**
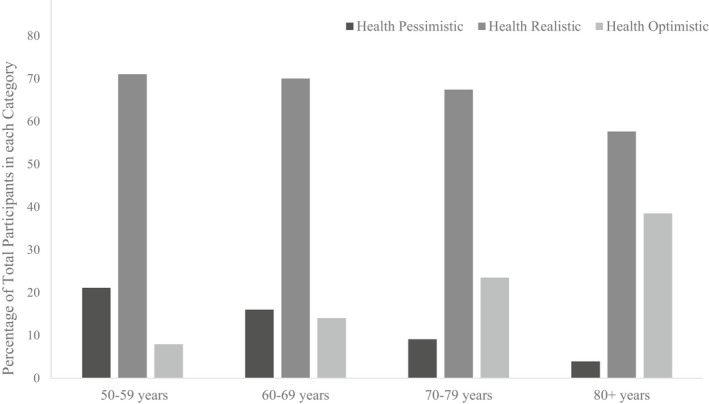
Comparing the percentage of participants in each health asymmetry category across age groups.

In total, 6,907 participants were included in the analysis, of which 45.84% of participants were male (*n* = 3,166), with all participants aged 50+ years: 50–59 years (*n* = 2,858), 60–69 years (*n* = 2,250), 70–79 years (*n* = 1,363), and 80+ years (*n* = 436). The health asymmetry metric was derived and prevalence rates for the health status categories were obtained: 16% of participants were health pessimistic (*n* = 1,104), 69.1% of participants were health realistic (*n* = 4,776), and 14.9% were health optimistic (*n* = 1,027). Most notably, the number of participants who were classified as health optimistic increased with older age groups, and conversely, the prevalence of health pessimistic individuals declined with an increase in age (see Table 3). Figure 1 below visualizes this change in prevalence rates for the health categories across all the age groups investigated. Descriptive statistics for the categorical and continuous variables were also tabulated (see Appendix).

**Table 3:** Valid percentages for the health rating categories across the various age groups

	Health pessimistic (*n* = 1,104)	Health realistic (*n* = 4,776)	Health optimistic (*n* = 1,027)
	Valid Percentage	Valid Percentage	Valid Percentage
50–59 years	21.1	1	7.9
60–69 years	16	70	14
70–79 years	9.1	67.4	23.5
80+ years	3.9	57.6	38.5

### Identifying predictors for health asymmetry categories

The full multinomial logistic model (χ^2^ (48, *N* = 6,907) = 6.45, *p* = .17) explained between 7.3% (Cox and Snell) and 9.1% (Nagelkerke) of the variance in health asymmetry status. Table 4 displays the results for likelihood of membership in the health pessimistic category. Males were less likely to be categorized as health pessimistic (OR = 0.62, *p* < .001). Those who were retired were significantly less likely to be classified as health pessimistic, when compared to those who were employed (OR = 1.28, *p* < .001) or unemployed (OR = 1.62, *p* < .001). The higher the educational attainment, the lesser likelihood of being categorized as health pessimistic: relative to those with no education, having a secondary level education was linked with a 18% lesser likelihood of being categorized here (OR = 0.82, *p* = .02), while those with a postgraduate degree had a 32% lesser likelihood of being categorized as health pessimistic (OR = 0.68, *p* = .02). The set of psychosocial variables yielded some significant associations with the health pessimistic category: a one‐point increase on the HADs anxiety scale was associated with a 3% increase in likelihood of being in health pessimistic (anxiety (OR = 1.03, *p* = .02). Similarly, a one‐point increase on the UCLA loneliness scale was associated with a 4% increased chance of being categorized as health pessimistic (OR = 1.04, *p* = .05). In terms of social connectedness scores, a one‐point increase was associated with a 13% lesser likelihood of receiving a health pessimistic classification (OR = 0.87, *p* = .003). Additionally, some health behaviour predictors elicited significant associations with health pessimism, including being a smoker (OR = 1.22, *p* = .003), engaging in regular vigorous exercise (OR = 1.34, *p* < .001), and occasional vigorous exercise (OR = 1.07, *p* < .001).

**Table 4:** Multinomial Logistic Regression analysis predicting likelihood of membership of the health pessimistic category

	*B*	*SE*	Wald	Sig.	OR (95% CI)
Health pessimistic					
Sociodemographic factors					
Sex (Ref = Female)	−.47	.08	−5.75	<.001	0.62 (0.53/0.73)
Marital status (Ref = Married)					
In a relationship	.06	.21	0.29	.77	1.06 (0.71/1.6)
Single	.13	.1	1.28	.20	1.14 (0.93/1.4)
Widowed	−.36	.13	−2.72	<.001	0.7 (0.54/0.9)
Education level (Ref = None)					
Secondary	−.2	.09	−2.35	.02	0.82 (0.7/0.97)
Certificate	−.17	.11	−1.50	.13	0.85 (0.68/1.05)
Undergraduate	−.3	.14	−2.17	.03	0.74 (0.56/0.97)
Postgraduate	−.39	.17	−2.32	.02	0.68 (0.49/0.94)
Hours of work per week	.0003	.002	0.17	.87	1 (1/1.01)
Work status (Ref = Retired)					
Employed	.25	.1	2.69	.01	1.28 (1.07/1.53)
Unemployed	.48	.1	5	<.001	1.62 (1.34/1.95)
Relative w/ Cancer diagnosis (Ref = No)	−.01	.08	−0.07	.94	0.99 (0.86/1.16)
Relative w/ Other serious illness (Ref = No)	.07	.08	0.92	.39	1.07 (0.92/1.25)
Psychosocial factors					
Intimate relationship	−.02	.07	−0.33	.74	0.98 (0.85/1.12)
Loneliness	.04	.02	2	.05	1.04 (1/1.07)
Social connectedness	−.14	.05	−2.95	.003	0.87 (0.79/0.95)
Anxiety	.02	.01	2.29	.02	1.03 (1/1.05)
Health behaviours					
Smoker (Ref = No)	.21	.07	2.85	.004	1.22 (1.07/1.42)
Alcohol consumption	−.02	.02	−1.06	.29	0.98 (0.94/1.02)
Vigorous exercise (Ref = Rare)					
Occasional	.07	.09	0.79	<.001	1.07 (0.90/1.28)
Regular	.29	.09	3.35	<.001	1.34 (1.13/1.59)
Sleep quality (Ref = Poor)					
Fair	.06	.13	0.48	.63	1.06 (0.83/1.35)
Good	−.02	.12	−0.42	.67	0.98 (0.78/1.24)
Cancer screening attendance (Ref = No)	−.01	.08	−0.17	.87	0.99 (0.85/1.15)

Note

B = unstandardized Beta value; OR (95% CI) = odds ratio with 95% confidence interval; Ref = reference category; *SE* = standard error for B; Sig = statistical significance; Wald = Wald chi‐square test.

In addition to predicting group membership of the health pessimistic category, predictions for the health optimistic category were also obtained (see Table 5). Males had a 50% increased likelihood of being health optimistic, in comparison to females (OR = 1.50, *p* < .001). Notably, being a widow was linked with a 72% greater likelihood of overestimating health status (OR = 1.72, *p* < .001). The other positively associated predictor of optimistic health perception was increased alcohol consumption (OR = 1.06, *p* = .01). Additionally, belonging to the health optimistic category was significantly and negatively associated with: being employed (OR = 0.39, *p* < .001) or unemployed (OR = 0.74, *p* < .001), relative to retirement. Finally, both occasional vigorous exercise (OR = 0.69, *p* < .001) and regular vigorous exercise (OR = 0.68, *p* < .001) were linked with a decreased likelihood of receiving a health optimistic classification.

**Table 5:** Multinomial Logistic Regression analysis predicting likelihood of membership of the health optimistic category

	*B*	*SE*	Wald	Sig	OR (95% CI)
Health optimistic					
Sociodemographic factors					
Sex (Ref = Female)	.41	.09	4.71	<.001	1.50 (1.27/1.78)
Marital status (Ref = Married)					
In a relationship	−.58	.31	−1.87	.06	0.56 (0.3/1.03)
Single	−.05	.12	−0.43	.67	0.95 (0.75/1.21)
Widowed	.54	.11	5.02	<.001	1.72 (1.39/2.12)
Education level (Ref = None)					
Secondary	−.11	.09	−1.33	.18	0.89 (0.75/1.06)
Certificate	.07	.11	0.62	.54	1.07 (0.86/1.34)
Undergraduate	−.04	.14	−0.32	.75	0.96 (0.73/1.25)
Postgraduate	−.21	.17	−1.19	.23	0.81 (0.58/1.14)
Hours of work per week	.003	.002	1.49	.14	1 (1/1.007)
Work status (Ref = Retired)					
Employed	−.93	.1	−9.65	<.001	0.39 (0.33/0.48)
Unemployed	−.31	.1	−3.38	<.001	0.74 (0.62/0.88)
Relative w/Cancer diagnosis (Ref = No)	−.03	.08	−0.32	.75	0.98 (0.84/1.13)
Relative w/other serious illness (ref = no)	.12	.08	1.42	.15	1.12 (0.96/1.32)
Psychosocial factors					
Intimate relationship	.04	.07	0.58	.57	1.04 (0.9/1.21)
Loneliness	−.03	.02	−1.39	.16	0.97 (0.94/1.01)
Social connectedness	.03	.05	0.63	.53	1.03 (0.94/1.14)
Anxiety	−.02	.01	−1.84	.07	0.98 (0.95/1)
Health behaviours					
Smoker (Ref = No)	−.03	.01	−0.40	.69	0.97 (0.84/1.12)
Alcohol consumption	.06	.02	3.29	<.001	1.06 (1.03/1.14)
Vigorous exercise (Ref = Rare)					
Occasional	−.37	.09	−4.38	<.001	0.69 (0.59/0.82)
Regular	−.39	.09	−4.32	<.001	0.68 (0.57/0.81)
Sleep quality (Ref = Poor)					
Fair	−.07	.13	−0.54	.59	0.93 (0.73/1.19)
Good	−.05	.12	−0.42	.68	0.95 (0.76/1.20)
Cancer screening attendance (Ref = No)	.05	.08	0.64	.52	1.05 (0.9/1.23)

Note

B = unstandardized Beta value; OR (95% CI) = odds ratio with 95% confidence interval; Ref = reference category; *SE* = standard error for B; Sig = statistical significance; Wald = Wald chi‐square test.

## Discussion

In this study, we created a useful health asymmetry measure that classifies older adults according to the discrepancy between their SRH and a more objective health status, as measured by the Frailty Index. This satisfied the primary aim of the study, as prevalence rates for these groups were obtained. While our results illustrate that a considerable majority of the study population were health realistic, a significant minority were found to exhibit discrepancies in their health assessment, being either health pessimistic or optimistic. Interestingly, the prevalence rate of health optimistic individuals increased incrementally with age, while, conversely, the prevalence of health pessimistic individuals decreased with age. This supports the claim that very advanced ageing can lead to the overestimation of healthiness, compared to younger groups (Henchoz et al., 2008).

The secondary aim of this study was to investigate potential associations between health rating discrepancies and a set of sociodemographic, psychosocial, and health behaviour variables. Meaningful results were obtained regarding the significant prediction of health asymmetry classifications, which helps fill a prevailing gap in the literature surrounding inaccuracies in SRH in ageing populations. Specifically, we found that some sociodemographic and health behaviour variables were relatively useful in predicting membership of the health pessimistic group. Females were more likely to be categorized here than males, which supports the SRH and frailty literature: females typically self‐report worse health than males (Idler, 2003), and tend to score higher on the FI (Gordon & Hubbard, 2020; Gordon et al., 2017). Generally, lower levels of education obtained led to a greater likelihood of being categorized as health pessimistic compared to those with no education. This is unsurprising as education has strong associations with health, self‐rated health (Volken, Wieber, Rüesch, Huber, & Crawford, 2017), and preventable mortality (Grytten, Skau, & Sørensen, 2020). However, associations between education and health must be interpreted with caution: educational attainment can often act as a proxy for socioeconomic status and wealth (Ware, 2019) and can be influenced by an individual’s ability to access health care (McMaughan, Oloruntoba, & Smith, 2020). Current smokers were more likely to be categorized as health pessimistic, providing underestimations of SRH, which aligns with findings from Wang et al. (2012). Though a noteworthy, and seemingly paradoxical, finding was that individuals who engage in more regular vigorous exercise were more likely to be categorized as health pessimistic relative to health realistic individuals. A potential explanation for this may be that individuals who assume that they are unhealthier may engage in physical exercise more than regular to combat this perceived ill health. This is also a finding which warrants further exploration.

Aside from the above sociodemographic and health behaviour measures, psychosocial factors – including loneliness, anxiety, and social connectedness – were also useful in predicting health pessimistic individuals. Associations between anxiety and underestimated SRH are perhaps not surprising here, given the intrinsic links between SRH and health anxiety (Hedman‐Lagerlöf et al., 2017). Therefore, there is merit in investigating whether health asymmetry could predict clinical outcomes, such as health anxiety, depression, or quality of life, for example. In addition, our study’s findings strengthen the link between loneliness and SRH: Nummela et al. (2011) found that little or no experience of loneliness yielded high SRH. Our findings show how higher levels of loneliness increase the probability of being categorized as health pessimistic, leading to an underestimation of SRH. Although nuanced theoretical differences exist between loneliness and social connectedness, being more socially connected also decreases the probability of being health pessimistic.

In contrast to the health pessimistic classification, the usefulness of the sociodemographic, psychosocial, and health behaviour variables became less defined with the health optimistic group. Males were more likely to be classified into the health optimistic group than females, which fits in line with existing SRH and frailty literature, discussed previously. Naturally, however, some major discrepancies exist between health pessimistic and health optimistic associations. Varying levels of educational attainment were not significant in predicting health optimistic individuals, unlike predicting health pessimistic individuals. This trend continues as no psychosocial variable was found to significantly predict membership of the health optimistic category. This is potentially explained by the lack of psychosocial impairment typically associated with health optimists. Though the most noteworthy finding from the health optimistic group is that increased levels of alcohol consumption were linked to an increased likelihood of being categorized as health optimistic, in comparison to health realistic. This adds to the counterintuitive body of literature of the associations between alcohol and SRH (Grønbæk et al., 1999; Theobald et al., 2003; Van Dijk et al., 2004).

However, there are limitations to this study and its design. The cross‐sectional design of the study is limiting, in that the associations between sociodemographic, psychosocial, and health behaviour variables with health asymmetry were at one specific time‐point: important longitudinal associations remain to be assessed. As discussed previously, the derivation of a categorical variable from continuous data comes at a cost: information is lost as the statistical power is reduced. Though, the benefits of categorizing at‐risk individuals were considered pertinent in this instance. Additionally, in constructing the FI, only 26 theoretically appropriate health deficits – measured in TILDA – were included in the analysis, whereas a minimum of 30 are recommended when deriving a FI (Searle et al., 2008). Although the FI was distributed as expected and FI scores increased with age, further investigation into health asymmetry should rectify this by including a minimum of 30 deficits. Additionally, the operationalization of alcohol consumption can be questioned, as the measure was ill‐defined. Alcohol consumption was measured based on how many days in a week a participant consumed alcohol, whereas it would have been more appropriate to measure the weekly consumption of alcohol units instead. Therefore, alcohol consumption associations are to be interpreted with caution. While the study’s general findings are relevant for the discrepancy of SRH and frailty in ageing populations, they cannot be generalized to all age groups. It must be flagged that the FI is not a suitable objective health measure for younger age groups, as the index compiles frailty deficits typically seen in advanced ageing populations, not younger ones. Health asymmetry needs to be applied to all age groups, using a different measure of objective health, to contrast SRH with. Since the FI computed age‐related deficits, age was not included as a factor in the multinomial logistic model; age is known to have a significant influence on the accuracy of SRH. Proceeding models should incorporate age, if alternative objective health measures can be found, instead of the FI.

There is potential for the discrepancies between SRH and frailty to be clinically meaningful. Nielsen (2016) argued that when individuals assess their own SRH that they are not only measuring their own global health status based on previous experiences, but also in anticipation of severe health events that are likely to occur to them in the future. As a result, the psychometric properties of the health asymmetry categorization should be investigated further, with the ultimate view to assess its utility in predicting health decline. Given the association between anxiety and health pessimism, there is potential for the health asymmetry metric to be useful in prediction of pre‐clinical and clinical health anxiety. Such investigation could utilize latent growth curve modelling to assess the clinical relevance of these health asymmetries, assessing the predictive ability of these categories longitudinally. In addition to testing health pessimistic categorization as a potential proxy for somatoform disorders, a further investigation into the link between health optimistic adults and quality of life would be of particular interest, for ageing populations. Further experimentation could assess how participants transition from one health category to another, across time. This would yield much needed evidence surrounding the rigidity of these classifications, and whether ageing individuals are likely to change group membership of the discrepancy between SRH and global health measures.

A novel and potentially clinically meaningful metric has been derived here, which creates categories based on the discrepancies between SRH and FI. The metric is a parsimonious and less burdensome way of identifying inaccurate health perceptions in ageing adults – a group of adults who are known to assess their health more inaccurately than their younger counterparts. This metric deals with the potential cognitive and emotional reporting tendencies of individuals (health optimistic and health pessimistic) and categorizes individuals accordingly. Based on the above findings, sociodemographic factors, psychosocial factors, and health behaviours play a useful role in the prediction of health pessimistic individuals. It may be useful to further assess the use of health asymmetry as a proxy for clinical constructs, as it may indicate those who are at‐risk for adverse physical health and mental health outcomes. Ultimately, this study provides further clarity on the identification of ageing individuals who – in terms of their health – may be expecting the best or fearing the worst.

## Conflicts of interest

All authors declare no conflict of interest.

## Author contribution

**Bill Calvey:** Conceptualization (equal); Formal analysis (equal); Methodology (equal); Visualization (equal); Writing – original draft (equal); Writing – review & editing (equal). **Joanna McHugh Power:** Conceptualization (equal); Methodology (equal); Supervision (equal). **Rebecca Maguire:** Conceptualization (equal); Methodology (equal); Supervision (equal); Writing – original draft (equal); Writing – review & editing (equal).

## Data Availability

The data that support the findings of this study are available upon request at https://www.ucd.ie/issda/data/tilda

## Appendix

Table A1 Descriptive statistics of continuous variables prior to imputation

**Table:**

	Mean	Std. error mean	Median	*SD*	Range
Loneliness	1.9	.03	1	2.16	0‐10
Anxiety	5.38	.05	5	3.6	0‐21
Social connectedness	2.88	.01	3	0.88	0‐4
Alcohol consumption	1.54	.02	1.5	1.91	0‐6.5
Hours of work per week	31.36	.3	35	14.15	0‐140
Frailty	31.33	.04	30.5	3.15	25–45

Table A2 Characteristics for the Health Asymmetry Categories for Ageing Individuals prior to imputation (*n* = 6907)

**Table:**

	Health Pessimistic (*n* = 1,104)		Health Realistic (*n* = 4,776)		Health Optimistic (*n* = 1,027)	
	Valid percentage		Valid percentage		Valid Percentage	
Sex			
Male	19.7		69		11.3
Female	12.9		69.3		17.8
Age			
50–59	21.1		71		7.9
60–69	16		70		14
70–79	9.1		67.4		23.5
80+	3.9		57.6		38.5
Marital status			
Married	15.7		70.7		13.6
In a Relationship	19.6		73		7.4
Single	21.5		66.8		11.7
Widowed	10.2		63.4		23.4
Employment status			
Retired	12.2		66.9		20.9
Employed	17.8		73.9		8.3
Unemployed	18.6		65.5		15.87
Educational level			
Primary	18.3		65.2		16.5
Secondary	15.9		70.5		13.6
Certificate	14.8		69.2		16
Undergraduate	13.2		71.7		15.1
Postgraduate	12.9		74.8		12.3
Relative w/ Cancer diagnosis			
Yes	15.6		69.5		14.9
No	16.1		69		15.9
Relative w/ Other serious illness			
Yes	16		68.8		15.2
No	15.9		70		14.1
Cancer screening participation			
Yes	15.2		69.7		15.1
No	17.8		68		14.2
Self‐rated health			
Excellent	0		62.3		37.7
Very Good	0.5		85.8		13.7
Good	22.1		69.1		8.8
Fair	39.3		51.8		8.9
Poor	40.5		53.2		6.3
Smoking status			
Smoker	18		67.6		14.4
Non‐smoker	13.4		71		15.6
Sleep quality			
Good	15.7		69.9		14.4
Fair	16.4		68.3		15.3
Poor	16.9		66.4		16.7
Intimate relationship			
Yes	14.4		68.4		17.2
No	17		69.6		13.4
